# Preservation of small extracellular vesicles for functional analysis and therapeutic applications: a comparative evaluation of storage conditions

**DOI:** 10.1080/10717544.2020.1869866

**Published:** 2021-01-11

**Authors:** Jun-Yong Wu, Yong-Jiang Li, Xiong-Bin Hu, Si Huang, Da-Xiong Xiang

**Affiliations:** a Department of Pharmacy, The Second Xiangya Hospital, Central South University, Changsha, China; b Hunan Provincial Engineering Research Center of Translational Medicine and Innovative Drug, Changsha, China; c Institute of Clinical Pharmacy, Central South University, Changsha, China

**Keywords:** Drug delivery, biodistribution, extracellular vesicles, exosomes, preservation

## Abstract

Extracellular vesicles (EVs) are nanovesicles involved in multiple biological functions. Small EVs (sEVs) are emerging as therapeutics and drug delivery systems for their contents, natural carrier properties, and nanoscale size. Despite various clinical application potentials, little is known about the effects of storage conditions on sEVs for functional analysis and therapeutic use. In this study, we evaluated the stability of sEVs stored at 4 °C, −20 °C, and −80 °C up to 28 days and compared them to fresh sEVs. Also, the effect of freeze-thawing circles on the quantity of sEVs was assessed. We found that different storage temperatures, along with shelf life, impact the stability of sEVs when compared to freshly isolated sEVs. Storage changes the size distribution, decreases quantity and contents, and impacts cellular uptake and biodistribution of sEVs. For functional studies, isolated sEVs are suggested to be analyzed freshly or stored at 4 °C or −20 °C for short-term preservation depending on study design; but −80 °C condition would be more preferable for long-term preservation of sEVs for therapeutic application.

## 1. Introduction

Extracellular vesicles (EVs) are cell-derived lipid bilayer-enclosed nanoscale vesicles. EVs are known for being able to work as natural vehicles to deliver components such as proteins and RNA from donor cells to recipient cells so that cells can communicate with their neighboring and distant cells (Tkach & Thery, 2016).

EVs have been emerging as attractive therapeutic tools for their content molecules generated from their parent cells and have gained great interest as delivery platforms due to their natural carrying ability (Garcia-Manrique et al., 2018). Exosomes, a subtype of EVs, are more attractive drug delivery vehicles for their relatively small size and properties such as crossing the biological barrier, circulation stability, and inherent targeting (Elsharkasy et al., 2020). While strategies are being developed to isolate different types of EVs, differential ultracentrifugation remains the most commonly used method for exosome separation and concentration (Thery et al., 2006). For the lack of specific markers of EV subtypes, it is suggested to describe EVs separated by ultracentrifugation (around 100,000 × *g*) as small EVs (sEVs) (Thery et al., 2018).

For therapeutic applications, sEVs are often obtained from cell culture. The collection of cell culture supernatant and differential ultracentrifugation-based exosomes isolation processes are time-consuming (Yang et al., 2020). However, little is known regarding how to store EVs before analyzing their contents, studying their functions, or for therapeutic applications. Generally, EVs are recommended to be stored at −80 °C (Jeyaram & Jay, 2017), but how storage condition affects the characteristics of EVs has not been fully elucidated and there is a lack of comparative evaluation of different storage conditions. Hence, toward successful clinical translation of sEVs, here we isolate bEnd.3 cells-derived sEV by differential ultracentrifugation and tested effects of storage conditions on size, quantity, and protein/RNA content and properties related to therapeutic applications of sEVs. Our data indicate that storage temperature affects the size, quantity, RNA/protein content, cellular uptake, and biodistribution of sEVs.

## 2. Materials and methods

### 2.1. Cell culture

Brain-derived Endothelial cell line bEnd.3 was maintained in Dulbecco’s modified Eagle’s minimum essential medium (DMEM) supplemented with 10% FBS and 1% penicillin/streptomycin/L-glutamine (Thermo Fisher Scientific, USA). Cells were incubated at 37 °C in humidified air with 5% CO_2_.

### 2.2. Isolation and characterization of sEVs

sEVs were prepared by differential centrifugation. The medium of bEnd.3 cells growing to 60% confluency (6 × 10^7^ cells) were replaced with EVs-depleted medium. 48 h after incubation, the supernatant was collected and then centrifuged at 300 × *g* for 10 min, 2000 × *g* for 10 min, 10,000 ×*g* for 30 min, and then filtered through a 0.2-μm filter. Afterward, the sEVs were pelleted by ultracentrifugation at 110,000 × *g* for 70 min and washed with phosphate-buffered saline (PBS) at 110,000 × *g* for 70 min then resuspended in PBS. All centrifugation process was performed at 4 °C within a day to obtain freshly isolated sEVs.

Images of sEVs fresh and after storage at different conditions for a week were observed by transmission electron microscopy (TEM). sEVs suspended in PBS were dropped onto the carbon film-coated copper grid and stained with 2% phosphotungstic acid. Images were captured using a Tecnai G2 Spirit TWIN Electron Microscope (FEI, Holland). The presence of protein markers CD63 (ab216130, Abcam, UK), TSG101 (ab125011, Abcam, UK), and Alix (sc53540, Santa Cruz Biotechnology, USA) on sEVs were detected via western blotting. Cell lysate and isolated sEVs were separated on SDS-PAGE gel and transferred onto PDVF membrane and analyzed using Amersham Imager 600 Imaging System.

### 2.3. Nanoparticle tracking analysis (NTA)

Since there has been a consensus that a low-temperature environment may be more suitable for storing sEVs, we thereby focusing on three common storage conditions: 4 °C, −20 °C, and −80 °C. Freshly isolated sEVs were aliquoted and separately stored at 4 °C, −20 °C, or −80 °C. Size distribution and concentration of sEVs fresh or after storage (3, 5, 7, 14, and 28 days) were analyzed using NTA (Nanosight NS300, Malvern, UK). Also, the effect of freeze-thawing circles (1–5 times) from −20 °C, −80 °C or liquid nitrogen to 4 °C on the quantity of sEVs was assessed and compared via NTA. Before performing NTA, samples were diluted by 20 times and resuspended in PBS. Samples were injected into Nanosight NS300 using a continuous syringe pump at an infusion rate of 20. The movement of nanoparticles under the camera was recorded and captured for 3 × 20 s. The detection threshold for nanoparticles was fixed at 3 for all tests.

### 2.4. Evaluation of contents in isolated sEVs

For contents in sEVs, we focus on two major contents: protein and RNA. Change of total protein level of sEVs after preservation was determined using a BCA Protein Assay Kit (MultiSciences Biotech Co., China). Change of level of tetraspanins CD63 in sEVs after preservation was evaluated using an enzyme-linked immunosorbent assay (ELISA) kit (CUSABIO Biotech Co. Ltd., China) according to the manufacturer’s instruction. RNA in sEVs after preservation was extracted using a Total Exosome RNA and Protein Isolation Kit (Invitrogen, USA) according to manufacturer’s instruction. Change of total RNA level of sEVs after preservation was evaluated using a Nanodrop 2000 spectrophotometer (Thermo Fisher Scientific, USA).

### 2.5. Cellular uptake study

To track sEVs in vitro, sEVs were labeled with PKH67 (green, Sigma-Aldrich, USA) as previously described (Li et al., 2020). For cellular uptake study, bEnd.3 cells were treated with PKH67 labeled autologous sEVs for 6 h, followed by fixing with paraformaldehyde (PFA) and staining with DAPI (Beyotime, China). Cellular uptake of PKH67-labeled sEVs in U87MG cells was observed using a confocal microscope (Leica TCS SP8 X, Leica, Germany).

### 2.6. Biodistribution study

To study the effect of storage conditions on the biodistribution of sEVs, healthy male BALB/c nude mice were employed as animal models. Isolated sEVs were labeled by carbocyanine dye DiR (Yeasen Biotechnology, China) for *in vivo* visualization (Li et al., 2020). Briefly, 10 μg of DiR was added to isolated sEVs fresh or after storage. After 20 min of incubation, unbounded DiR dye was removed by ultracentrifugation. DiR-labeled sEVs was resuspended in PBS. 100 μL of DiR-labeled sEVs were administrated to mice through tail vein injection and fluorescence was obtained using an AniView100 multimodal imaging system (Biolight Biotechnology Co., Ltd., China) at different time points. *Ex vivo* biodistribution was inspected after in vivo biodistribution monitoring. The animal study was carried out using the Institutional Animal Care and Use Committee (IACUC)-approved procedures. Animals were purchased from SJA Laboratory Animal Co., LTD (Hunan, China) and housed according to the regulations of the IACUC.

### 2.7. Statistical analysis

Data were presented as mean values ± SD. Student’s *t*-test was performed at the significance level of *α* = 0.05 to evaluate differences between groups.

## 3. Results

### 3.1. Characterization of sEVs

**Figure 1.**
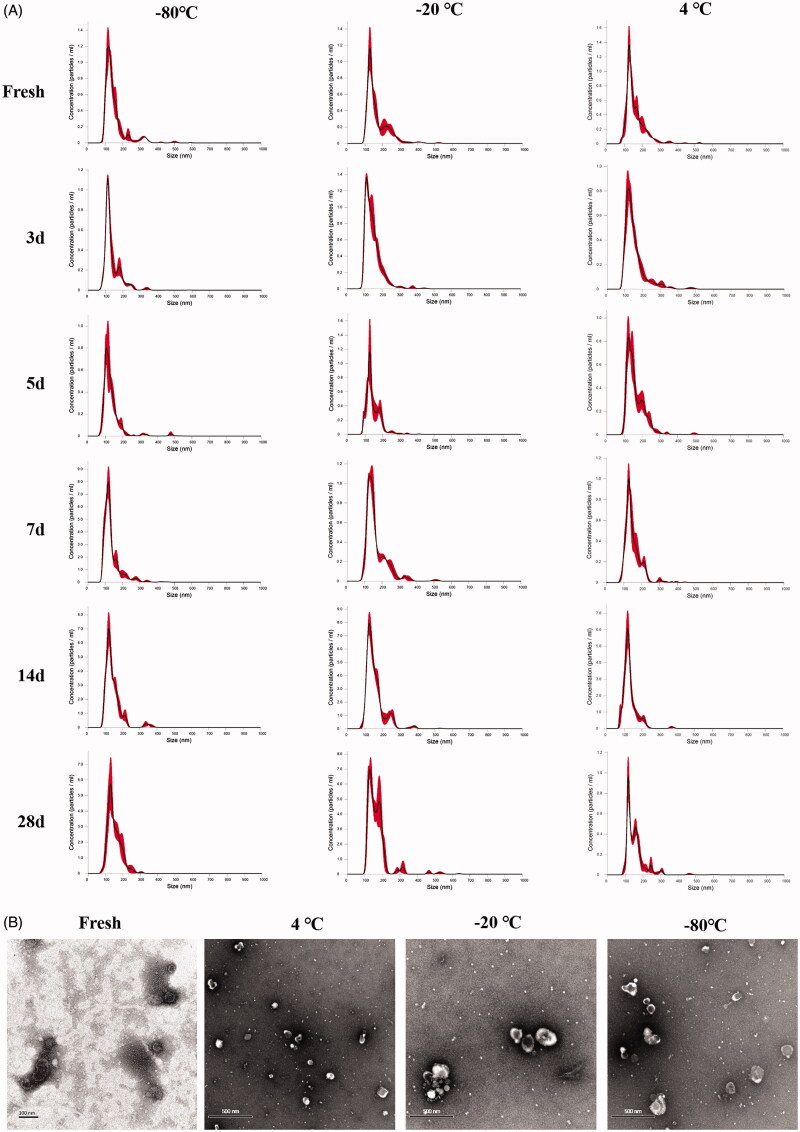
Characterization of sEVs under different storage conditions. (A) NTA graph of sEVs. (B) TEM images of sEVs.

Isolated sEVs were characterized by size distribution, TEM, and protein markers. The enrichment of sEVs markers, CD63, TSG101, and Alix was identified via western-blotting (Figure S1). The size distribution of fresh sEVs by NTA (Figure 1(A)) was matched as observed under TEM (Figure 1(B)). Images of sEVs under TEM after storage at different temperatures all showed the presence of sEVs, but 1-week storage would cause significant aggregations (Figure 1(B)).

### 3.2. NTA of sEVs

**Figure 2.**
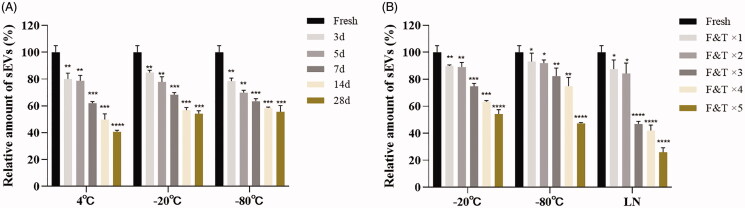
Effects of storage condition and freeze-thawing circles on quantity of sEVs. (A) Relative quantity of sEVs after storage at different conditions. (B) Relative quantity of sEVs after freeze-thawing circles under different temperature. Data were presented as Mean ± SD. *p < .05; **p < .01; ***p < .001. LN: Liquid nitrogen; F&T: Freeze-thawing.

sEVs fresh or stored up to 28 days at different temperatures all exhibited fine size distribution by NTA (Figure 1(A)). However, further analysis of particle quantity showed that the number of sEVs decreased quickly after storage for all conditions. −20 °C and −80 °C slowed the rate of decrease in nanoparticle numbers, but there was still a more than 40% loss of sEVs particle after 28 days of storage (Figure 2(A)). Freeze-thawing also impacted significantly the number of sEVs. Freeze-thawing in liquid nitrogen seriously damaged sEVs, and running freeze-thawing circles between −20 °C/−80 °C 4 °C also contributed significantly to the loss of sEVs particles (Figure 2(B)).

**Figure 3.**
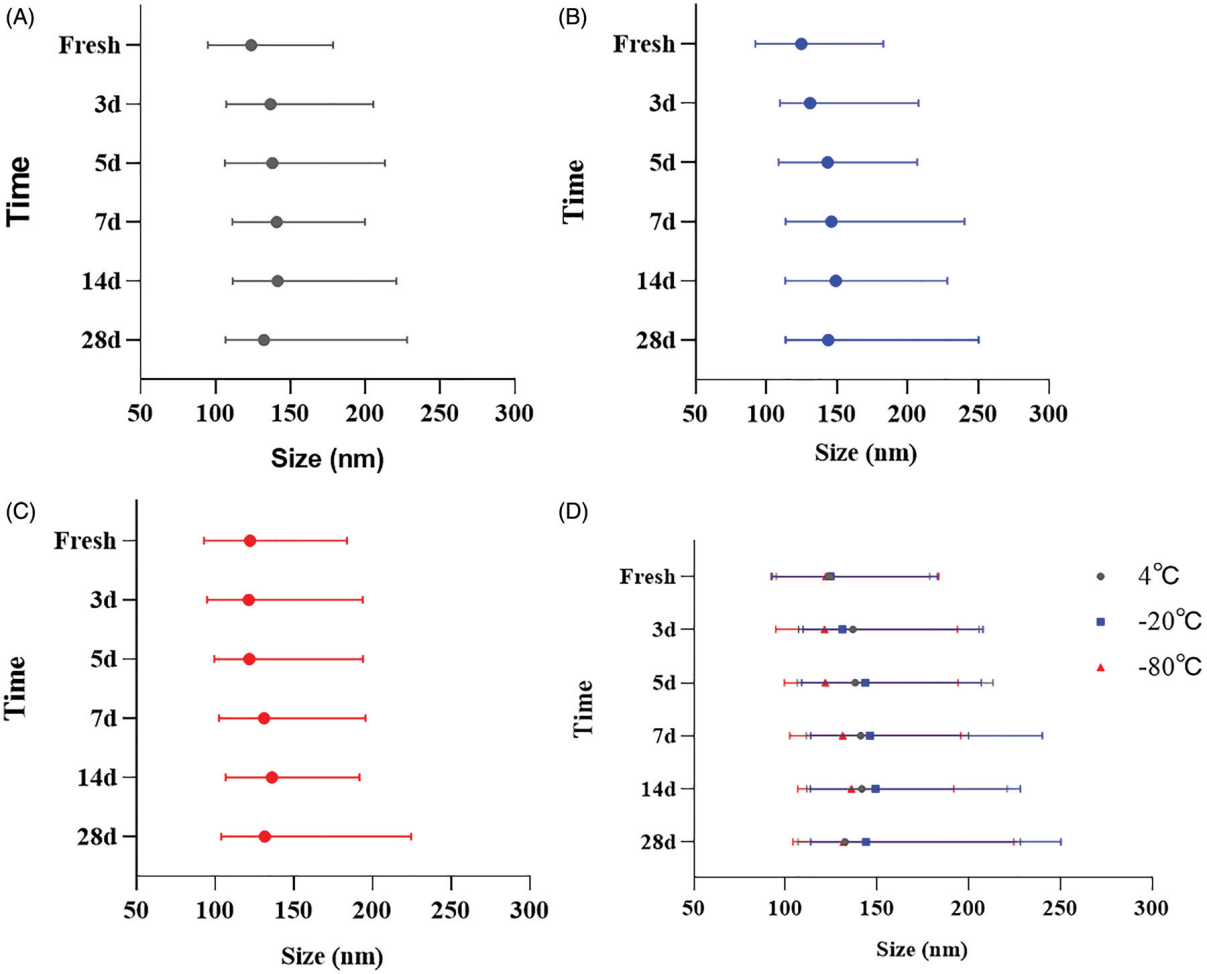
Cumulative size distribution of sEVs under different storage conditions. (A) Change of size distribution of sEVs stored at 4 °C. (B) Change of size distribution of sEVs stored at −20 °C. (C) Change of size distribution of sEVs stored at −80 °C. (D) Summary of change of size distribution of sEVs stored at different conditions. Bar shows the size range from D10 to D90, symbol shows the D50.

**Figure 4.**
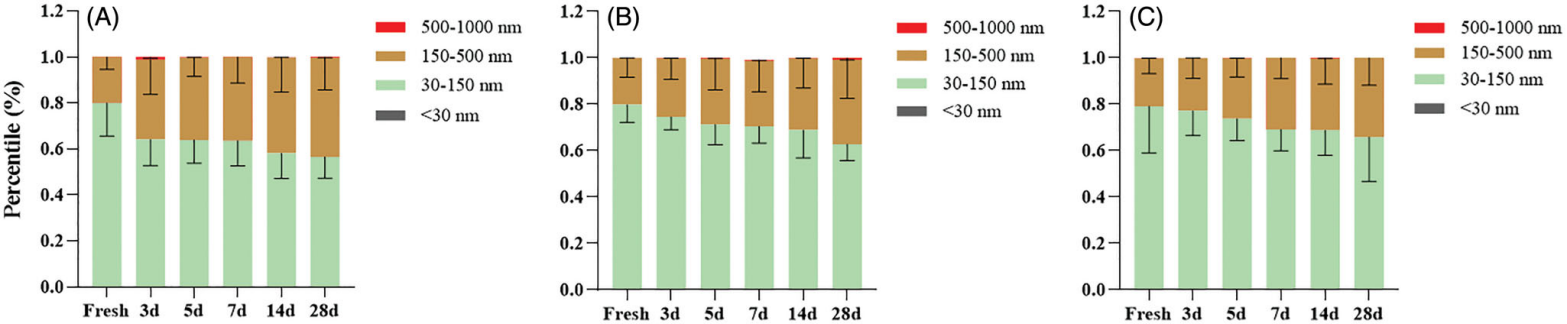
Size distribution of sEVs under different storage conditions (A) 4 °C; (B) −20 °C; (C) −80 °C. Data were presented as Mean ± SD.

Results of the analysis of cumulative size distribution showed that the size range from D10 to D90 of sEVs was widened for all storage conditions (Figure 3), but −20 °C enlarged the size more remarkably (Figure 3(D)). Similarly, a decreasing trend by the time of percentage of small particles (30–150 nm) in isolated sEVs was observed for all storage conditions, along with an increased percentage of large particles (150–500 nm) due to the loss of small particles (Figure 4).

### 3.3. Contents in sEVs

**Figure 5.**
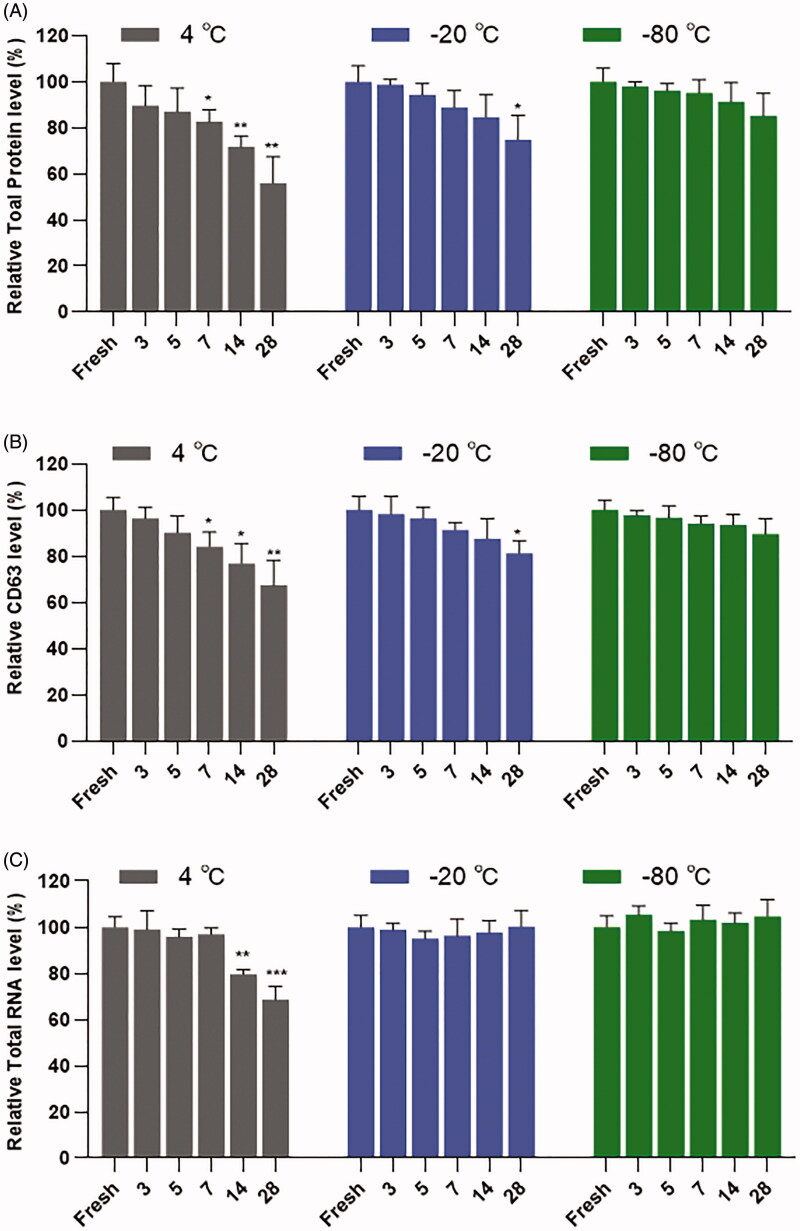
Analysis of contents in sEVs under different storage conditions. (A) Relative total protein levels in sEVs. (B) Relative CD63 levels in sEVs. (C) Relative RNA levels in sEVs. All tests were repeated three times. Data were presented as Mean ± SD. *p < .05; **p < .01; ***p < .001.

BCA test of total protein level showed that the storage of sEVs at 4 °C resulted in decreased protein levels after a week, however, the storage of sEVs at −80 °C showed no significant decrease of protein level during 28 days of preservation (Figure 5(A)). Consistent with the total protein level, there was a sharp decrease of CD63 in sEVs at 4 °C, but not at −80 °C during 28 days of preservation. Besides, a significantly slowed decreasing trend of CD63 was observed at −20 °C (Figure 5(B)). RNA in sEVs was more stable than protein during preservation. There was no significant decrease in total RNA in sEVs at 4 °C conditions within a week. We did not observe the loss of total RNA in sEVs at −20 °C or −80 °C during 28 days of preservation (Figure 5(C)).

### 3.4. Cellular uptake

**Figure 6.**
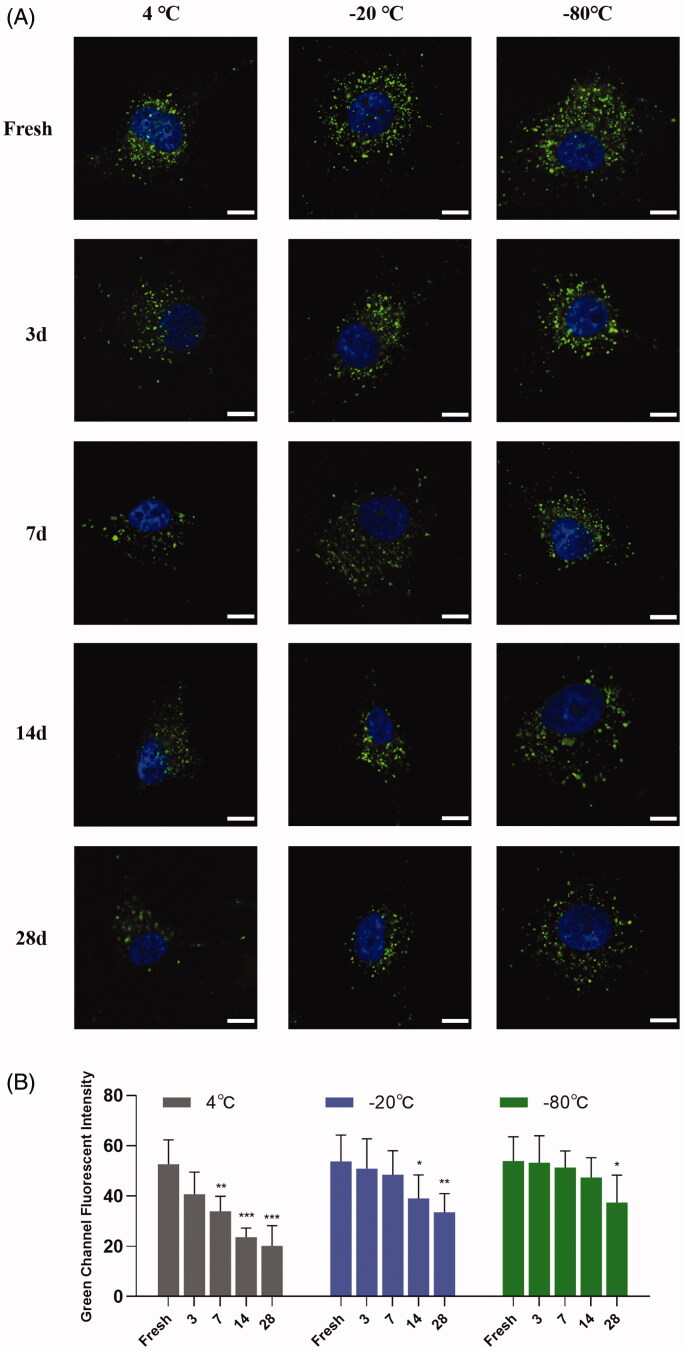
Cellular uptake of sEVs under different storage conditions. (A) Autologous cellular uptake of bEnd.3 cells derived sEVs. Images were obtained 4 h after incubation. Scale bar = 100 μm. (B) Semi-quantitative analysis of autologous cellular uptake of bEnd.3 cells derived sEVs. *p < .05; **p < .01; ***p < .001.

Storage conditions influenced cellular uptake of sEVs along with shelf life. Storage of sEVs at 4 °C showed significantly decreased autologous cellular uptake efficiency; however, the uptake efficiency remained as highly as fresh for sEVs preserved at −80 °C within three weeks. Also, there was a decreasing trend of uptake efficiency for sEVs preserved at −20 °C, but the difference became significant after 14 days of storage (Figure 6).

### 3.5. Biodistribution

**Figure 7.**
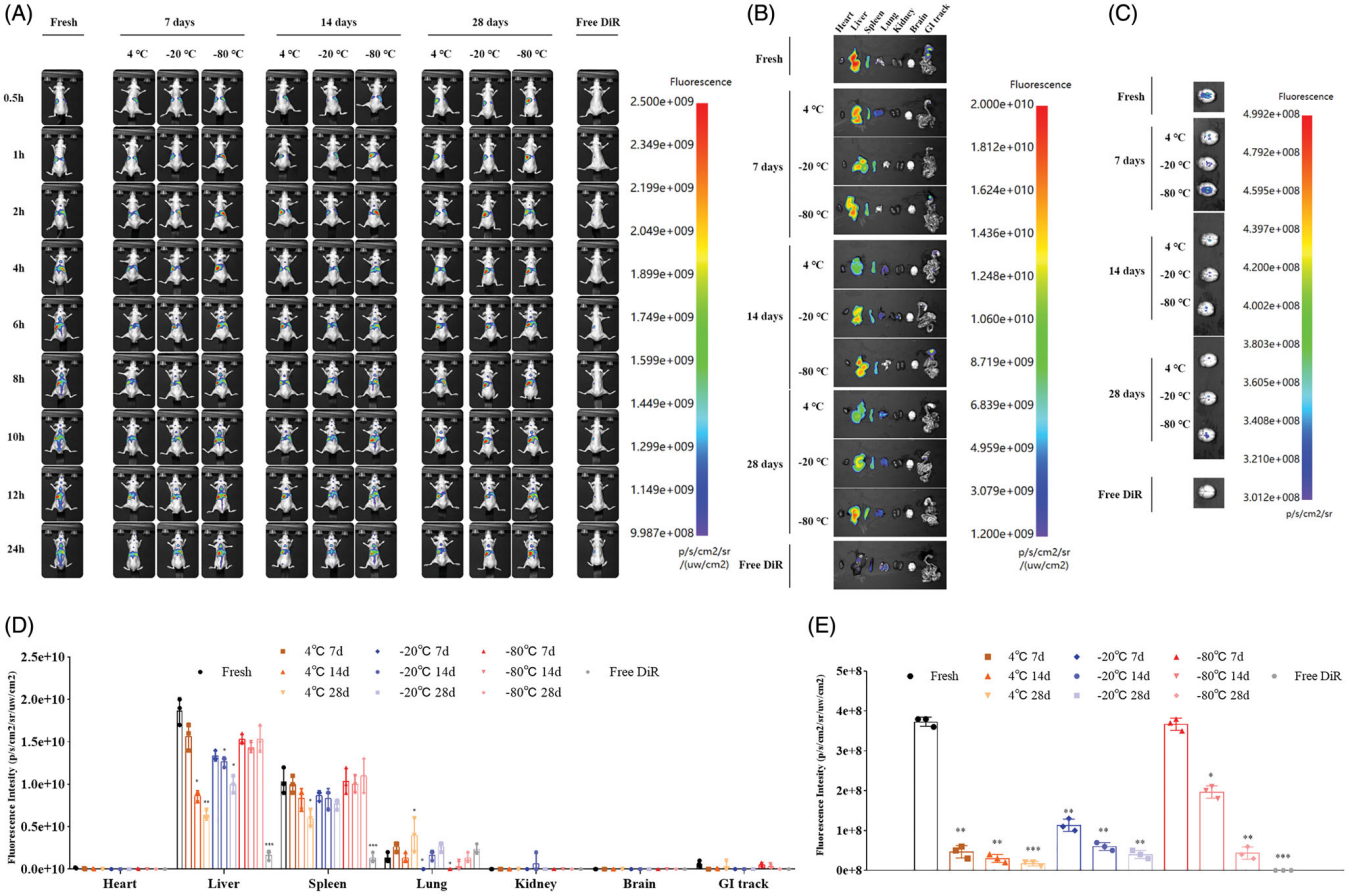
Biodistribution of sEVs (A) *In vivo* biodistribution of DiR-labeled sEVs fresh and after storage at different conditions intravenously administered to healthy mice. (B) *Ex vivo* biodistribution of major organs in mice receiving DiR-labeled sEVs fresh and after storage at different conditions. (C) Comparative biodistribution of brain in mice receiving DiR-labeled sEVs. (D) Intensity of fluorescence signals in various organs of mice receiving DiR-labeled sEVs. (E) Intensity of fluorescence signals in brains of mice receiving DiR-labeled sEVs. *p < .05; **p < .01; ***p < .001 (compared to fresh sEVs).

Healthy mice were administrated with DiR-labeled sEVs fresh or after storage through tail vein injection and imaged at different time points to monitor biodistribution. Similar to our results of contents in sEVs and cellular uptake, fresh sEVs showed strong fluorescence signals in the whole body, *ex vivo* organs, and brain (Figure 7). For storage at 4 °C or −20 °C, we observed significantly decreased fluorescence signals of sEVs with shelf life (Figure 7(A)). The fluorescence signals can hardly be detected in gastrointestinal (GI) tracks (Figure 7(B,D)) as well as in brains (Figure 7(C,E)). For storage at −80 °C, we observed stable fluorescence signals in mice and in *ex vivo* organs within 28 days of storage (Figure 7(A,B)). However, fluorescence signals in brains were significantly decreased after 14 days of storage (Figure 7(C,E)).

## 4. Discussion

EVs have tremendous potentials for therapeutic applications. For clinical application of EV-based biopsy or therapy, storage conditions are supposed to have minimal impact on EV integrity, contents and functions. In this study, we investigated the effects of storage temperature and shelf life on properties of sEVs related to therapeutic use. We found that freshly isolated sEVs showed the best results in all tests. Different storage temperatures, along with shelf life, affect the stability of sEVs and their functions to varying degrees.

International Society for Extracellular Vesicles (ISEV) recommends storage of isolated EVs in phosphate-buffered saline at −80 °C (Witwer et al., 2013), but more data is required for supporting the consensus. There have been several studies exploring favorable temperature for EVs storage, with inconsistent results. Sokolova et al. reported that the storage of exosomes derived from three different cell types (HEK 293 T, ECFC, MSC) at −20 °C and freezing-thawing circles up to 10 times have minimal effect on size by NTA (Sokolova et al., 2011). In contrast, Lee et al. reported that −70 °C was the favorable condition for HEK293 cells-derived exosomes isolated using the Exo-Quick kit for long-term storage for basic researches as there was less significant loss of exosomal protein and RNA compared to room temperature and at 4 °C after 10 days of storage (Lee et al., 2016). In another study, Maroto et al. investigated the effects of storage temperature on the stability of airway exosomes, they found that 4 °C and −80 °C storage conditions for four days both affect the proteomic content of exosomes and suggested immediate analysis of exosomes for diagnostic and functional studies (Maroto et al., 2017). Similar to their results, Cheng et al. isolated HEK 293 T cells-derived exosomes by using the ExtraPEG method and investigated storage conditions on quantity and cellular uptake of exosomes (Cheng et al., 2019). They reported that storage at 4 °C had the highest exosome concentration and exosomal protein levels for short-term storage (24 h); however, for long-term storage (over a week), exosomes showed the best stability when stored at −80 °C.

Our data revealed that the optimal storage condition for sEVs may vary depending on the study purpose. Results of NTA produced acceptable size distribution graphs of sEVs during 28 days of storage for all storage temperatures (Figure 1(A)). However, TEM results demonstrated aggregation of sEVs after a week of storage at all temperatures (Figure 1(B)). Further analysis revealed that storage temperature, along with shelf life, decreased significantly the quantity of sEVs (Figure 2). Freezing-thawing should be avoided as it damaged sEVs seriously. Storage of sEVs increased cumulative size distribution, especially at −20 °C (Figure 3), but there was a notable loss of 30–150 nm particles for sEVs stored at 4 °C (Figure 4). Therefore, for integrity and quantity, sEVs are supposed to be stored at −80 °C avoiding freezing-thawing, but short-term storage (within a week) at 4 °C is also acceptable. For contents in sEVs, total protein and CD63 levels in sEVs decreased sharply at 4 °C and the difference becomes significant after a week of storage. However, the total RNA level in sEVs started to decrease after 14 days of storage at 4 °C. It is likely that the 4 °C environments maintained the integrity of sEVs during the first week thus protected the RNA content. In contrast, we observed no significant decrease of RNA level for sEVs stored at −20 °C or −80 °C (Figure 5). Therefore, for studies focusing on contents and functions, sEVs may be more suitable to be stored at −20 °C or −80 °C.

An important application of sEVs is as therapeutics or to be engineered as drug delivery systems. For therapeutic use, storage seems to be inevitable. Although a previous study reported that storage temperature did not influence the cellular uptake of exosomes considering the loss in quantities, the shelf life (not reported) may be too short to observe differences (Cheng et al., 2019), there has been a lack of standardized criterion of preservation condition of sEVs and little is known regarding impacts of storage temperature and shelf life on properties of sEVs as vehicles *in vitro* nor *in vivo*. In our study, we found that cellular uptake of sEVs decreased significantly soon after storage at 4 °C, while cellular uptake of sEVs stored at −20 °C or −80 °C was relatively stable within 14 days. Given that storage at −20 °C or −80 °C decreased the number of sEVs significantly but not total protein or CD63 levels, it is possible that the decreased cellular uptake of sEVs (stored at 4 °C) has resulted from the loss of their protein contents.

It has been reported that bEnd.3 cells-derived exosomes can cross the blood–brain barrier and enter the brain (Yang et al., 2015, 2017). The intensity of fluorescence signal in the brain may be an indicator reflecting their stability at different storage temperatures for therapeutics applications. Mice receiving fresh sEVs showed the strongest fluorescence signals in the brain (Figure 7). Of all the storage temperature and shelf life tested, only sEVs stored at −80 °C for a week showed high fluorescence intensity in the brain (Figure 7(C)), suggesting that storage influence significantly the brain targeting ability of bEnd.3 cells-derived sEVs. Hence, for therapeutic applications of sEVs, it is supposed to be used as soon as possible or can be stored at −80 °C for short-term preservation.

Inconsistency in EVs isolation, characterization, and analysis limited the comparability between studies investigating storage effects on EVs. Isolation methods, affect the feasibility, yield, and purity of EVs (Shtam et al., 2018). Studies using commercial EVs isolation kit without technical details may have limited reproducibility for future studies, and materials may affect downstream profiling or functional analysis of EVs. In this regard, we thus used the most common differential ultracentrifugation method to isolate sEVs to provide a practical reference for future studies. Besides, it has been reported that the detection method influences the results of the characterization of EVs (Almizraq et al., 2017). Methods in previous studies may be inconsistent in the characterization and analysis of EVs and reduce the comparability. Future studies following standardized methods, such as those recommended by ISEV (Witwer et al., 2013; Thery et al., 2018), would aid progress in the field.

Aside from preserving isolated sEVs directly after resuspending in PBS, lyophilization, a common method for preservation of thermolabile materials (Assegehegn et al., 2020), has been used to preserve EVs for analysis (Stamer et al., 2011; Lydic et al., 2015) and to produce EVs formulations (Bari et al., 2019). Lyophilization can extend the shelf life of EVs and freeze-dried EVs may be stored directly at room temperature (Charoenviriyakul et al., 2018) to reduce cost. However, those studies are preliminary and lack standard protocols (Kusuma et al., 2018). The choice of appropriate cryoprotectant for sEVs preservation also requires further investigation.

## 5. Conclusion

In conclusion, our study provided relatively comprehensive information on the effects of storage conditions on sEVs in regards to their further functional analysis and therapeutic applications. To accelerate the clinical translation of sEVs, detailed storage protocols are warranted. Furthermore, the development of novel preservation methods is encouraged to increase the commercial availability of sEVs in the future.

## Supplementary Material

Supplemental Material
