# Supplementary figures and images for: Preservation of small extracellular vesicles for functional analysis and therapeutic applications: a comparative evaluation of storage conditions
Source: Drug Deliv. 2021 Jan 11;28(1):162–70. doi: 10.1080/10717544.2020.1869866 (PMC7808382; doi:10.1080/10717544.2020.1869866)

**Supplementary material**


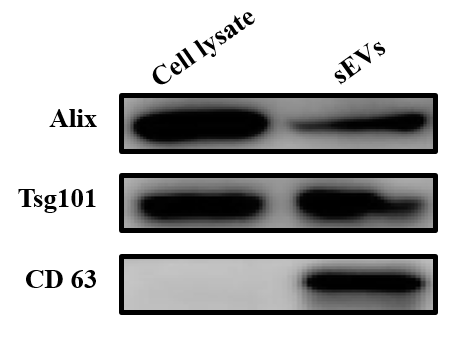


Figure S1. Western blot of sEVs isolated from bEnd.3 cells.

Supplement: Supplemental Material [file IDRD_A_1869866_SM9282.docx]
